# Short-Course High-Intensity Statin Treatment during Admission for Myocardial Infarction and LDL-Cholesterol Reduction—Impact on Tailored Lipid-Lowering Therapy at Discharge

**DOI:** 10.3390/jcm13010127

**Published:** 2023-12-25

**Authors:** Víctor Marcos-Garcés, Héctor Merenciano-González, María Luz Martínez Mas, Patricia Palau, Josefa Inés Climent Alberola, Nerea Perez, Laura López-Bueno, María Concepción Esteban Argente, María Valls Reig, Raquel Muñoz Alcover, Inmaculada Pradillas Contreras, Ana Arizón Benito, Alfonso Payá Rubio, César Ríos-Navarro, Elena de Dios, Jose Gavara, Francisco Javier Chorro, Juan Sanchis, Vicente Bodi

**Affiliations:** 1 Department of Cardiology, Hospital Clinico Universitario de Valencia, 46010 Valencia, Spain; hectormeren@gmail.com (H.M.-G.); mluzmmas@comv.es (M.L.M.M.); patricia.palau@uv.es (P.P.); mvallsr@gmail.com (M.V.R.); raquelalcover@hotmail.com (R.M.A.); inmapracon@yahoo.es (I.P.C.); francisco.j.chorro@uv.es (F.J.C.); sanchis_juafor@gva.es (J.S.); 2 INCLIVA Health Research Institute, 46010 Valencia, Spain; neere_8@hotmail.com (N.P.); cesar.rios@uv.es (C.R.-N.); 3 Department of Medicine, Faculty of Medicine and Odontology, University of Valencia, 46010 Valencia, Spain; 4 Department of Rehabilitation, Hospital Clinico Universitario de Valencia, 46010 Valencia, Spain; inescliment093@gmail.com (J.I.C.A.); laura.lopez@uv.es (L.L.-B.); lulilloluli@yahoo.es (M.C.E.A.); paya_alf@gva.es (A.P.R.); 5 Hospital Clinico Universitario de Valencia, 46010 Valencia, Spain; arizon_anaben@gva.es; 6 Network Biomedical Research Center for Cardiovascular Diseases (CIBER-CV), 28029 Madrid, Spain; elenaddll@gmail.com; 7 Centre for Biomaterials and Tissue Engineering, Universitat Politècnica de València, 46022 Valencia, Spain; jose_4_6_90@hotmail.com

**Keywords:** high-intensity statin, lipid-lowering therapy, low-density lipoprotein cholesterol, acute myocardial infarction, cardiac rehabilitation

## Abstract

We hypothesized that a short-course high-intensity statin treatment during admission for myocardial infarction (MI) could rapidly reduce LDL-C and thus impact the choice of lipid-lowering therapy (LLT) at discharge. Our cohort comprised 133 MI patients (62.71 ± 11.3 years, 82% male) treated with atorvastatin 80 mg o.d. during admission. Basal LDL-C levels before admission were analyzed. We compared lipid profile variables before and during admission, and LLT at discharge was registered. Achieved theoretical LDL-C levels were estimated using LDL-C during admission and basal LDL-C as references and compared to LDL-C on first blood sample 4–6 weeks after discharge. A significant reduction in cholesterol from basal levels was noted during admission, including total cholesterol, triglycerides, HDL-C, non-HDL-C, and LDL-C (−39.23 ± 34.89 mg/dL, *p* < 0.001). LDL-C levels were reduced by 30% in days 1–2 and 40–45% in subsequent days (R^2^ 0.766, *p* < 0.001). Using LDL-C during admission as a reference, most patients (88.7%) would theoretically achieve an LDL-C < 55 mg/dL with discharge LLT. However, if basal LDL-C levels were considered as a reference, only a small proportion of patients (30.1%) would achieve this lipid target, aligned with the proportion of patients with LDL-C < 55 mg/dL 4–6 weeks after discharge (36.8%). We conclude that statin treatment during admission for MI can induce a significant reduction in LDL-C and LLT at discharge is usually prescribed using LDL-C during admission as the reference, which leads to insufficient LDL-C reduction after discharge. Basal LDL-C before admission should be considered as the reference value for tailored LLT prescription.

## 1. Introduction

In recent decades, therapeutic advances both in acute and chronic phase have allowed for a sustained increased in survival and better prognosis in acute myocardial infarction (MI) patients [1]. However, survivors after the acute phase, which currently account for most MI sufferers, continue to have an increased risk of adverse cardiovascular events at short- and long-term follow-up [2,3]. This fact effectively classifies them as individuals with very high cardiovascular risk [4], and, like all patients with established cardiovascular disease, intensive therapy is required to achieve secondary prevention goals. 

Cholesterol, and specifically low-density lipoprotein cholesterol (LDL-C), has been recognized not only as a relevant risk factor but also as the main causal factor of the atherosclerotic processes that underlie most cases of cardiovascular disease [5,6]. Thus, the main goal of lipidic prevention after a MI is to achieve and maintain LDL-C < 55 mg/dL and a ≥50% reduction from previous LDL-C levels [4,7,8,9]. 

For this instance, it is recommended that a high-intensity statin is initiated as soon as possible in MI sufferers after admission and that a lipid profile is obtained during admission, preferably within 24 h of presentation [9,10,11]. Theoretically, knowing the basal lipid profile of the patient will allow for tailored lipid-lowering therapy (LLT) after discharge, choosing the most appropriate drug or drug combination in order to reach LDL-C goals. However, many MI patients undergo lipid profile analysis > 24 h after admission. Given that even a short-course treatment with a high-intensity statin such as atorvastatin can induce a fast and significant reduction in LDL-C levels [12], the choice of LLT after discharge may be inappropriate if guided by a lipid profile that has been drawn under the effects of this therapy. 

In our study, we aim to define the effect, in a real-world setting, of a short-term course of a high-intensity statin treatment (oral atorvastatin 80 mg o.d.) during admission for MI to reduce LDL-C levels, and to study the influence of this potential LDL-C reduction compared to basal LDL-C levels in the choice of appropriate LLT at discharge. 

## 2. Materials and Methods

### 2.1. Population

**Figure 1:**
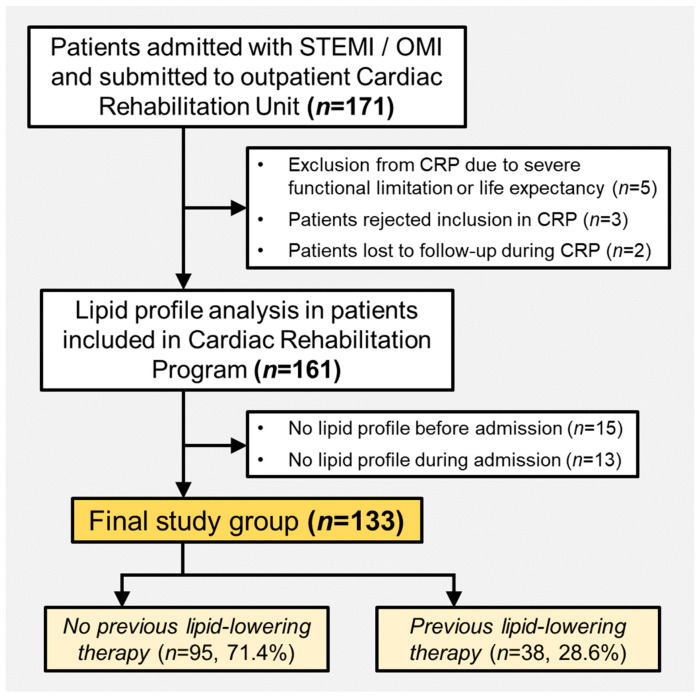
Flowchart of patients included in the study. Abbreviations: CRP = Cardiac Rehabilitation Program. OMI = occlusion myocardial infarction. STEMI = ST-segment elevation acute myocardial infarction.

The study population comprised all patients admitted in our institution, a high-complexity tertiary care hospital, for ST-segment elevation myocardial infarction (STEMI) or occlusion myocardial infarction (OMI), i.e., those without persistent ST-segment elevation but other signs of acute coronary occlusion (e.g., persistent chest pain and left or right bundle branch block, transitory ST-segment elevation, or hyperacute T waves). Patients were referred to the Cardiac Rehabilitation Program (CRP) from January 2022 and October 2023. A total of 5 patients were excluded from the CRP due to severe functional limitation or life expectancy, 3 patients voluntarily rejected inclusion in the CRP, and 2 patients were lost to follow-up after inclusion in the CRP. After inclusion, we registered the lipid and metabolic profile analyzed during admission and prior to admission, if available. Some patients were excluded from the analysis due to unavailable lipid and metabolic profiles before (*n* = 15) or during admission (*n* = 13). The final study group comprised 133 patients. The patient flowchart is shown in Figure 1. 

We registered baseline clinical characteristics such as age, sex, cardiovascular risk factors, infarct location, Killip class during admission, Global Registry of Acute Coronary Events (GRACE) risk score, and echocardiographic left ventricular ejection fraction (LVEF) before discharge. 

### 2.2. Cardiac Rehabilitation Program

In our institution, STEMI and OMI patients are referred to the Cardiac Rehabilitation Program after hospital discharge. Follow-up visits are provided in a specific outpatient unit by a multidisciplinary team of cardiologists, Physical Medicine and Rehabilitation physicians, trained nurses, and physiotherapists. After initial clinical stabilization, conventional or cardiopulmonary exercise testing (C/CPET) is performed, patient risk for CRP is stratified (as low, intermediate, or high risk), and an individualized aerobic and strength training program is prescribed, including ambulatory training for at least 3 months and in-hospital supervised training in selected cases for at least 1 to 2 months. During this Phase 2 of CRP, pharmacological therapy is modified at the discretion of the cardiologists in charge of patients to achieve optimal control of cardiovascular risk factors, complete smoking cessation, and other adjustment of cardiological drugs when needed (e.g., antianginal therapy or prognostic and symptomatic therapy for heart failure patients). At the end of Phase 2, C/CPET is repeated, updated training guidance for Phase 3 is provided, and achievement of control goals is analyzed (cardiovascular risk factors, quality of life assessment, etc.). 

### 2.3. Lipid and Metabolic Profile Analysis

We registered the lipid and metabolic profile of the cohort before and during hospital admission. For the baseline lipid and metabolic profile, the electronical clinical history record was revised, and the most recent blood test was registered. Patients underwent a blood test with a complete lipid and metabolic profile during admission, which was also registered. Some patients were excluded due to unavailable lipid and metabolic profiles before or during admission (see flowchart in Figure 1). Additionally, the lipid and metabolic profile was analyzed on first blood sample performed 4 to 6 weeks after hospital discharge. 

The following variables were studied: fasting glucose (mg/dL), glycated hemoglobin (HbA1c, in %), total cholesterol (mg/dL), triglycerides (mg/dL), HDL cholesterol (HDL-C, mg/dL), LDL-C (mg/dL), and non-HDL-C (mg/dL). Additionally, lipoprotein (a) levels were analyzed during admission. 

### 2.4. Corrected LDL-C and Potency of LLT

To analyze the potency for LDL-C reduction in each LLT (Table S1), we used data from the National Institute for Health and Care Excellence (NICE) guidelines [13], the University of Michigan (UMHS) Lipid Therapy Guideline [14], the American 2018 Guideline on the Management of Blood Cholesterol [15], the Cochrane Collaboration [16,17,18], Spanish real-life registries [19], and several clinical trials, reviews and information from real clinical practice [20,21,22,23,24,25,26]. If no specific data were found regarding an exact combination of drugs, we used the formula for multiple percentage changes [27], which reads:

Final LDL-C with LLT = Initial LDL-C × (1 − % reduction with drug #1) × (1 − % reduction with drug #2) × (1 − % reduction with drug #3) × (1 − % reduction with drug #4)

To calculate the percentage of reduction in LDL-C that a combined treatment would induce, we used the following formula [27]:

% reduction in LDL-C with LLT = [1 − (final LDL-C/initial LDL-C)] × 100

In the subset of patients treated with LLT before admission (*n* = 38, 28.6%), we calculated the corrected basal LDL-C by an extrapolation using their measured LDL-C levels before admission and the estimated potency of the LLT which they were being treated with at that time. We used the following formula:

Corrected basal LDL-C = LDL-C with LLT/1 − (% reduction in LDL-C with LLT/100)

### 2.5. LLT and LDL-C Reduction during Admission

Following our institution’s acute coronary syndrome protocol, all patients were treated with oral atorvastatin 80 mg o.d. during admission, irrespective of their previous ambulatory LLT, if any. 

We calculated the absolute differences in all lipid and metabolic variables compared to levels before admission. Specifically for LDL-C levels, the absolute (in mg/dL) and relative (in %) reduction during admission were calculated, and comparisons were made in patients with and without LLT before admission. Measured LDL-C levels and corrected basal LDL-C levels were used as a reference. 

We calculated the absolute (in mg/dL) and relative (in %) reduction in LDL-C according to days of admission, i.e., days from admission to blood test extraction during which patients were treated with oral atorvastatin 80 mg o.d.

### 2.6. LLT at Discharge and LDL-C Levels during Follow-Up

We registered the LLT which was prescribed to each patient at discharge by referring cardiologists. Using the theoretical percentage reduction in LDL-C which each LLT should induce, we calculated the theoretical LDL-C level that each patient would achieve. Two models were performed: the first one using real LDL-C levels measured at admission (which we theorized cardiologists were using as a reference for prescribing LLT at discharge) and the second one using corrected basal LDL-C levels before admission. Patients were then categorized according to whether they would theoretically reach the LDL-C < 55 mg/dL target or not, and the two models were compared. 

We also analyzed the real measured LDL-C levels at the first blood test during follow-up (4 to 6 weeks after hospital discharge) and compared the percentage of patients achieving the LDL-C < 55 mg/dL target. 

### 2.7. Objectives of the Study

The main objectives of our study were as follows:

1. To define the efficacy, in a real-world setting, of a short-term course of a high-intensity statin treatment (oral atorvastatin 80 mg o.d.) during admission for MI to reduce LDL-C levels.
2. To study the influence of this potential LDL-C reduction compared to basal LDL-C levels in the choice of appropriate LLT at discharge.

### 2.8. Ethics

The study was conducted in accordance with the Declaration of Helsinki and approved by the Ethics Committee for Drug Research (CEIm) of Hospital Clinico Universitario de Valencia (protocol code: 2019/262 and date of approval: 26 May 2020). Informed consent was obtained from all subjects involved in the study. 

### 2.9. Statistical Analysis

The one-sample Kolmogorov–Smirnov test was used to test normal data distribution. For continuous parametric variables, data are expressed as mean ± standard deviation and were analyzed with Student’s *t* test. Continuous non-parametric variables are shown as median plus interquartile range and compared with the Mann–Whitney U test. Qualitative variables are presented as percentages and compared using the chi-square test or Fisher’s exact test. To compare lipid and metabolic variables before and after admission, Student’s t test for paired samples was used. To calculate the absolute and percentage reduction in LDL-C levels according to days from admission to blood test extraction, we computed a lineal regression analysis and ANOVA test. Histograms of theoretical LDL-C achieved with LLT at discharge were analyzed using LDL-C at admission and corrected basal LDL-C before admission as a reference, and also of real measured LDL-C 4 to 6 weeks after hospital discharge. Statistical significance was considered for 2-tailed *p*-values < 0.05. The SPSS statistical package version 26.0 was used.

## 3. Results

### 3.1. Cohort Description

The final cohort comprised 133 STEMI/OMI patients discharged from our hospital, included in our local CRP, and in whom lipid and metabolic profiles were available before and during admission. Baseline characteristics and lipid and metabolic profile variables are depicted in Table 1. 

Mean age was 62.71 ± 11.3 years, most patients were male (*n* = 109, 82%), and hypercholesterolemia was the most prevalent cardiovascular risk factor (*n* = 124, 93.2%), although only 28.6% of patients (*n* = 38) received LLT before admission. Anterior infarction was the most prevalent infarct location (*n* = 69, 51.9%). The risk profile during admission was defined by a mean GRACE risk score of 117.66 ± 29.81 points, 39 (29.3%) patients were categorized as Killip class ≥ 2, mean LVEF was 52.07 ± 10.6%, and 51 (38.3%) had LVEF < 50%. 

### 3.2. Previous LLT and Lipid Profile before Admission

**Figure 2:**
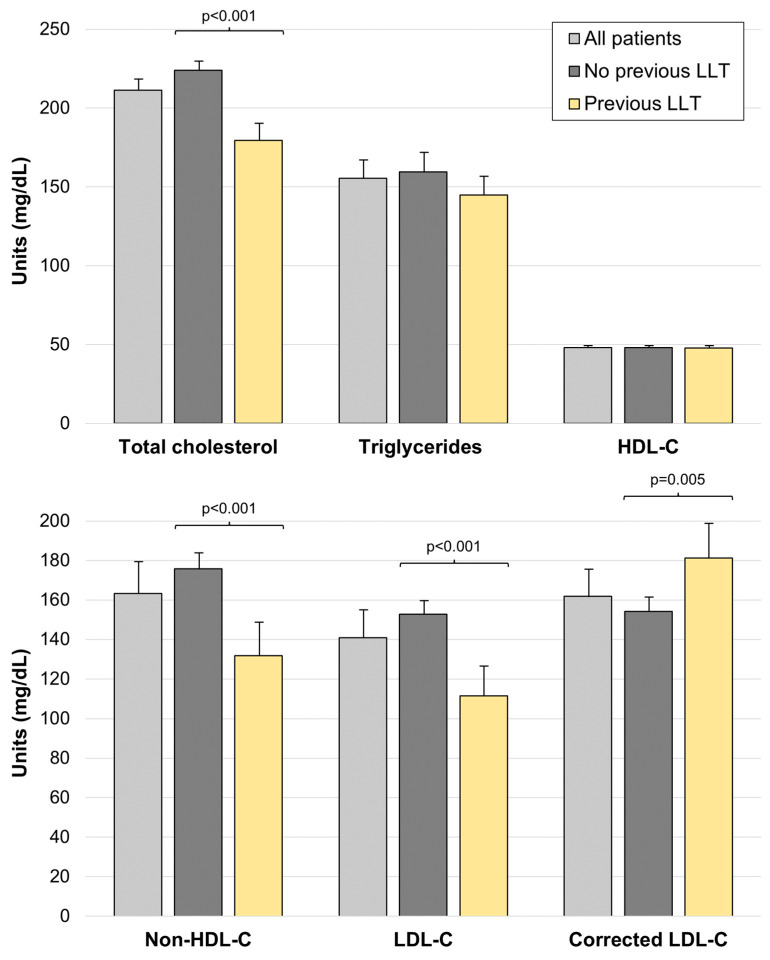
Lipid profile before admission in patients with and without previous LLT. Abbreviations: HDL-C = high-density lipoprotein cholesterol. LDL-C = low-density lipoprotein cholesterol. LLT = lipid-lowering therapy.

Lipid profile before admission (Table 1, Figure 2) was remarkable for relatively elevated total cholesterol (211.32 ± 50.9 mg/dL), non-HDL-C (163.28 ± 48.54 mg/dL) and LDL-C (140.93 ± 41.26 mg/dL) levels. In patients undergoing LLT before admission, all these levels were significantly lower than in patients not on lipid-lowering medications (total cholesterol: 175.82 ± 47.2 vs. 225.52 ± 45.26 mg/dL, non-HDL-C: 128.11 ± 43.46 vs. 177.35 ± 43.19 mg/dL, and LDL-C: 108.63 ± 38.67 vs. 153.85 ± 34.8 mg/dL, all comparisons *p* < 0.001). However, after correcting LDL-C levels for previous LLT to calculate the basal LDL-C, corrected LDL-C was higher in patients with previous LLT (181.96 ± 55.44 vs. 153.85 ± 34.8 mg/dL, *p* = 0.005), indicating higher basal LDL-C levels, which may have prompted preventive intervention by clinicians. 

Patients undergoing previous LLT were also older (67.44 ± 10.7 vs. 60.82 ± 11 years, *p* = 0.002), less frequently male (68.4% vs. 87.4%, *p* = 0.01), and depicted a higher prevalence of cardiovascular risk factors such as hypertension (71.1% vs. 54.7%, *p* = 0.08) and diabetes mellitus (42.1% vs. 15.8%, *p* = 0.001).

### 3.3. Lipid Profile before and during Admission

Blood tests for the lipid and metabolic profile analysis were obtained at a median of 3 [1,2,3,4] days after admission. Patients were treated with oral atorvastatin 80 mg o.d. for the same number of days. Significant differences were noted in the lipid and metabolic profile during admission compared to basal levels before admission (Table 2). Fasting blood glucose and HbA1c increased significantly and similarly in patients with and without previous LLT. 

During admission, a significant reduction in cholesterol from basal levels was noted in the cohort, including total cholesterol (−49.21 ± 43.01 mg/dL, *p* < 0.001), triglycerides (−17.41 ± 79.25 mg/dL, *p* = 0.01), HDL-C (−8.52 ± 8.82 mg/dL, *p* < 0.001), non-HDL-C (−40.69 ± 41.4 mg/dL, *p* < 0.001), and LDL-C (−39.23 ± 34.89 mg/dL, *p* < 0.001). Notably, the magnitude of LDL-C reduction was greater when compared to basal LDL-C levels corrected by LLT before admission (−60.18 ± 40.22 mg/dL, *p* < 0.001).

Compared to patients with previous LLT, patients without previous LLT experienced a significantly higher reduction in total cholesterol (−60.55 ± 41 vs. −20.87 ± 34.27 mg/dL, *p* < 0.001 for comparison), non-HDL-C (−52.07 ± 39.03 vs. −12.24 ± 32.87 mg/dL, *p* < 0.001 for comparison), and LDL-C levels (−48.76 ± 34.24 vs. −15.4 ± 23.4 mg/dL, *p* < 0.001 for comparison) during admission. On the contrary, when compared to basal corrected LDL-C levels, patients with previous LLT depicted a significantly higher reduction in LDL-C during admission (−88.72 ± 40.28 vs. −48.76 ± 34.24 mg/dL, *p* < 0.001 for comparison).

### 3.4. Days of Admission and LDL-C Reduction

**Figure 3:**
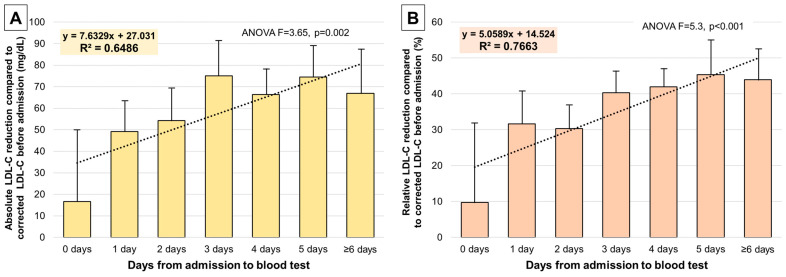
LDL-C reduction during admission compared to corrected basal LDL-C before admission according to days from admission to the blood test. (**A**): Absolute LDL-C reduction (in mg/dL). (**B**): Relative LDL-C reduction (in %). Lineal regression with the corresponding formula and R^2^ values are shown. Patients were treated with oral atorvastatin 80 mg o.d. Abbreviations: ANOVA = analysis of variance. LDL-C = low-density lipoprotein cholesterol. R^2^ = R-squared.

We calculated the absolute (in mg/dL) and relative (in %) difference in LDL-C levels from corrected basal LDL-C according to the days from admission to blood test extraction, and concurrent treatment with atorvastatin 80 mg o.d. (Figure 3). A significant absolute and relative reduction in LDL-C from basal levels was noted, and a significant correlation existed with days of admission until the blood test and the magnitude of LDL-C reduction (*p* = 0.002 and *p* < 0.001, respectively). When the blood test was sampled at day 1 or 2 after admission, reductions of approximately 30% in LDL-C levels were noted, which accounted for 40–45% in subsequent days. Regression analyses with corresponding formulas are depicted in Figure 3. 

### 3.5. LLT at Discharge and LDL-C Levels during Follow-Up

LLT at discharge was registered (Table 3). Most patients (98.5%) were discharged on statin treatment, and high-intensity statins such as atorvastatin and rosuvastatin were frequently selected. Ezetimibe was prescribed in approximately half of patients (52.6%) at discharge. The use of proprotein convertase subtilisin/kexin type 9 (PCSK9) inhibitors and fibrates was marginal (0.8% and 2.3%, respectively). 

**Figure 4:**
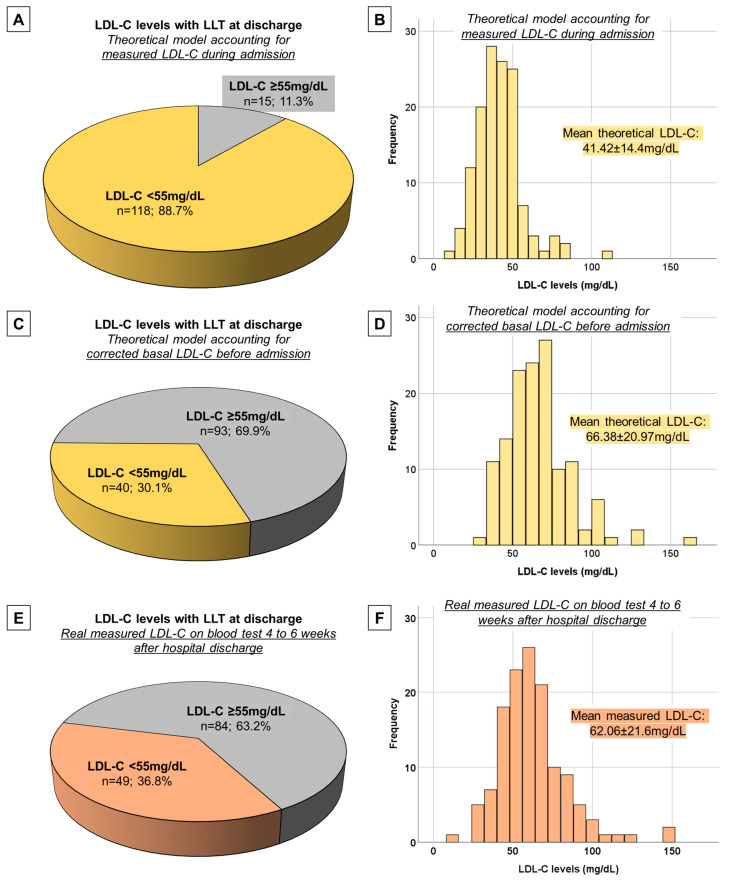
Theoretical LDL-C levels with LLT at discharge according to LDL-C during admission and corrected basal LDL-C, compared to real measured LDL-C after discharge. In the model accounting for real measured LDL-C during admission, most patients would theoretically achieve the LDL-C < 55 mg/dL target (**A**), and the mean theoretical LDL-C is lower (histogram in (**B**)). However, in the model accounting for corrected basal LDL-C, only a minority of patients would theoretically achieve target LDL levels (**C**), and the mean theoretical LDL-C is above 55 mg/dL (histogram in (**D**)). This second model is more consistent with the real measured LDL-C levels on the first blood test 4 to 6 weeks after hospital discharge (**F**) and the proportion of patients achieving the LDL-C target (**E**). Abbreviations: LDL-C = low-density lipoprotein cholesterol. LLT = lipid-lowering therapy.

We considered the estimated potency for LDL-C levels reduction for each LLT as previously described and calculated the theoretical LDL-C levels than each patient would achieve with the LLT prescribed at discharge. When LDL-C levels at admission were used as the reference value, we noticed that most patients (88.7%) will theoretically achieve the LDL-C target of <55 mg/dL with the prescribed LLT at discharge (Figure 4). However, if corrected basal LDL-C levels were considered as the reference value, only a small proportion of patients (30.1%) will theoretically achieve this lipid target with the prescribed LLT at discharge (Figure 4). The mean theoretical LDL-C after LLT at discharge was higher in the model that considered corrected basal LDL-C than in the model that considered LDL-C during admission (66.38 ± 20.97 mg/dL vs. 41.42 ± 14.4 mg/dL, *p* < 0.001). 

We also analyzed LDL-C levels on the first blood test after discharge, which was sampled at a median of 4 [3,4,5,6] weeks. Real measured LDL-C levels (mean 62.06 ± 21.6 mg/dL) were concordant with the theoretical model accounting for corrected basal LDL-C, and the proportion of patients achieving target LDL-C < 55 mg/dL was also very similar to this model (36.8%). 

## 4. Discussion

The main relevant points of our study are that: (1) A short course of atorvastatin 80 mg o.d. administered per protocol during admission for MI can induce a rapid and significant reduction in LDL-C from pre-admission levels. (2) The absolute and relative reduction in LDL-C is related to days of treatment, accounting for approximately 30% in the first two days and 40–45% in subsequent days. (3) LLT at discharge is probably selected based on admission LDL-C levels, which will theoretically achieve target LDL-C in most patients. (4) However, accounting for corrected basal LDL-C, only a minority of patients achieve target LDL-C with this treatment, both in a theoretical model and in real measured LDL-C 4 to 6 weeks after hospital discharge. 

### 4.1. Lipid Management and Control after MI

LDL-C has been recognized as the main causal factor of the atherosclerotic processes that underlie most cases of MI [5,6], and thus it is mandatory to maintain low LDL-C levels after the index event to reduce subsequent cardiovascular risk. MI sufferers are considered individuals with very high cardiovascular risk, and a secondary prevention target of LDL-C < 55 mg/dL plus a ≥50% reduction from previous LDL-C levels is recommended [4,7,8,9]. Available evidence suggests that each reduction of 38 mg/dL (1 mmol/L) in LDL-C levels can achieve a reduction of 22–25% in major adverse cardiovascular events [28,29]. 

Fortunately, a diverse and growing therapeutic armamentarium for lowering LDL-C is available to the clinician, and in recent years several drugs have been incorporated that can lower LDL-C as much as 85% in combination therapy [30,31]. High-intensity statin treatment with either atorvastatin or rosuvastatin is systematically recommended after an MI [9,10,11], given that they further reduce cardiovascular risk compared to lower-intensity statins [32] and that other statins could hardly achieve the required LDL-C target. The addition of other agents such as ezetimibe or iPCSK9 are recommended in patients who remain uncontrolled despite previous LLT, but economic, social, and healthcare barriers exist, and variability in prescription is common in daily clinical practice. 

Unfortunately, optimal LDL-C control in real-life is very infrequent. In the latest EUROASPIRE V survey in 131 centers in 81 regions of 27 European countries, only 29% of patients achieved an LDL-C target of <70 mg/dL, which was the goal for secondary prevention at that time [33]. The percentage of patients with LDL-C < 55 mg/dL was even lower. In a more contemporary survey in 2018 to 2775 patients in 7 European countries, only 17% achieved LDL-C < 70 mg/dL in the first ambulatory follow-up visit and 32% in the second one [34]. Interestingly, the increased prescription of LLT on top of statin therapy correlated with better lipidic control. These results are concerning and emphasize the need for strategies to improve lipidic control in patients after MI [35]. 

### 4.2. Strategies from Lipidic Control after MI

As discussed, the first strategy that can enhance lipidic control after MI is the generalized use of high-intensity statin therapy. It is recommended that a high-intensity statin is initiated as soon as possible in MI sufferers after admission to increase patient adherence after discharge and to rapidly and steadily improve cardiovascular outcomes [9,10,11]. In our hospital, we use oral atorvastatin 80 mg o.d. per protocol in patients admitted for MI. To this regard, the trend in recent years is very positive and shows a preferential prescription of high-intensity statins in MI patients, compared to 2016–2017, when roughly half of patients were treated with this high-intensity LLT [36,37,38]. 

The use of add-on LLTs such as ezetimibe has gained relevant attention in recent years after the IMPROVE-IT trial [39], which showed a significant reduction in LDL-C levels and improved cardiovascular outcomes when ezetimibe was added to statin therapy. Several authors even advocate for initial combined LLT with a high-intensity statin plus ezetimibe after MI, in a paradigm shift from “high-intensity statin therapy” to “high-intensity lipid-lowering therapy” [27,40]. A structured follow-up after MI using this strategy has been shown to reach an optimal LDL-C target in 80% of patients, increasing to 100% if bempedoic acid or iPCSK9 were sequentially added when needed [41]. In selected patients with very high LDL-C levels, the early use of iPCSK9 could also be justified [40]. 

Optimization of LLT is vital for lipidic control, but proper clinical care pathways must also be considered a priority. For instance, Cardiac Rehabilitation Programs are the ideal framework to provide patients not only with close follow-up and LLT optimization but also with structured multidisciplinary intervention in healthy dietary habits, exercise prescription, and therapeutic adherence, among others [42,43]. Even if a patient is not included in a Cardiac Rehabilitation Program after MI, a blood test should be drawn, and a follow-up visit should be scheduled 4 to 6 weeks after hospital discharge, with subsequent LLT optimization if lipidic targets are not reached [7,15,44]. However, real-life registries show that the rate of lipid testing after MI is disappointingly low in many countries, and, when performed, it is usually obtained 3 or more months after hospital discharge [45,46]. 

All these data emphasize the need for tailored, individualized LLT at discharge after a MI, effective enough to rapidly and steadily reduce LDL-C and allow for optimal lipid control in subsequent months and years. 

### 4.3. Lessons Learned: Optimization of LLT at Discharge

Undoubtedly, lipid control is of utmost importance to clinical cardiologists who care for admitted MI sufferers. Thus, we hypothesized that in our center LLT at discharge was being carefully selected. 

In our cohort, the use of high-intensity statins at discharge was nearly universal, and ezetimibe was prescribed in approximately half of patients, probably in line with current clinical practice in most hospitals with similar characteristics. Interestingly, we showed that most patients (nearly 90%) would theoretically achieve target LDL-C < 55 mg/dL with LLT prescribed at discharge if lipid levels during admission were considered as a reference. This is probably a strong indicator that cardiologists are using lipid profile during admission, specifically LDL-C, as the reference to select the most appropriate LLT at discharge. 

However, when patients were visited in our Cardiac Rehabilitation Program, we noticed that target LDL-C levels were rarely achieved on the first ambulatory blood test 4 to 6 weeks after discharge. In fact, if basal LDL-C levels were considered as a reference (after correcting for previous LLT, if any), less than a third of patients (30.1%) would theoretically reach target LDL-C. This percentage was much closer to the real LDL-C levels that we were registering, which in fact were 36.8% on the first ambulatory blood test. We hypothesized that lipid profile during admission may be altered by oral atorvastatin with which patients were being treated throughout their hospital stay. Moreover, a spontaneous reduction in cholesterol concentrations has been noted during the acute setting of a MI, likely attributable to inflammatory and acute phase reactions, adrenergic-mediated adipocyte lipolysis, and dietary changes [47,48,49]. Thus, this combination of factors can affect the lipid profile during admission. 

As suspected, a significant reduction in lipid levels, specifically in LDL-C, was noted during admission as compared to pre-admission levels, and a significant and positive correlation existed between days from admission to blood sampling and the absolute and relative LDL-C reduction. For instance, a 30% reduction in LDL-C was noted as early as the first or second day after admission, and a 40–45% reduction was registered in following days. 

The fact that high-intensity statin therapy during admission rapidly and significantly lowers LDL-C can have a dual interpretation. Firstly, it is bad news that lipid profile during admission for MI does not accurately reflect basal cholesterol levels, given that it is not obtained before any high-intensity statin administration. Moreover, clinicians seem to consider these cholesterol levels as a reference to prescribe LLT at discharge, which turns out to be, in most instances, insufficient. On the other hand, in patients presenting with MI who had never had a lipid test before, our study provides some guidance to extrapolate basal LDL-C values using LDL-C levels during admission and days of treatment with high-intensity statin (in this case, oral atorvastatin 80 mg o.d.). 

The main implications for clinical practice are that LLT at discharge after MI should be individualized according to basal LDL-C, defined as previous LDL-C levels in LLT-naïve patients, or corrected LDL-C levels if patient was receiving any LLT at that time. In patients with unavailable LDL-C levels before admission, basal LDL-C should be extrapolated from the admission blood test accounting for a 30–45% reduction in LDL-C if treatment with high-intensity statin is being administered. These recommendations could help achieve early and sustained LDL-C targets via tailored and effective LLT at discharge. 

### 4.4. Study Limitations

Our study has several limitations that should be discussed. Firstly, it derives from a single center, which may not be representative of the clinical reality in other centers and countries. Secondly, the cohort is relatively small, although homogeneous and with defined and structured management during admission and follow-up. Third, the correction for basal LDL-C levels in patients with previous LLT before admission may have some inaccuracies. Finally, it would be desirable to confirm these results in prospective studies specifically designed to improve LLT prescription at discharge according to basal LDL-C levels. Until then, the impact of implementing these recommendations in LDL-C control after MI remains speculative. 

## 5. Conclusions

We conclude that, in a real-world scenario, a short-course treatment with high-intensity statin during admission for MI can induce a rapid and substantial reduction in cholesterol levels, specifically LDL-C, which correlates with days of treatment. Our results also suggest that LLT at discharge is usually prescribed using LDL-C during admission as the reference, which leads to insufficient LDL-C reduction in both a theoretical model and real measured LDL-C 4 to 6 weeks after hospital discharge. Basal LDL-C before admission should be considered as the reference value for tailored LLT prescription at discharge and optimized lipid control after MI.

## Figures and Tables

**Table 1:** Baseline characteristics and lipid and metabolic profiles of patients included in the Cardiac Rehabilitation Program.

	All Patients (*n* = 133)	No Previous LLT (*n* = 95)	Previous LLT (*n* = 38)	*p*-Value
**Clinical variables**				
Age (years)	62.71 ± 11.3	60.82 ± 11	67.44 ± 10.7	0.002
Male sex (%)	109 (82)	83 (87.4)	26 (68.4)	0.01
Hypercholesterolemia (%)	124 (93.2)	86 (90.5)	38 (100)	0.04
Hypertension (%)	79 (59.4)	52 (54.7)	27 (71.1)	0.08
Diabetes mellitus (%)	31 (23.3)	15 (15.8)	16 (42.1)	0.001
Smoking habit (%)	65 (48.9)	48 (50.5)	17 (44.7)	0.55
Killip class ≥ 2 (%)	39 (29.3)	25 (26.3)	14 (36.8)	0.23
GRACE risk score	117.66 ± 29.81	114.27 ± 29.22	126.27 ± 29.93	0.04
Anterior infarction (%)	69 (51.9)	51 (53.7)	18 (47.4)	0.51
LVEF (%)	52.07 ± 10.6	51.41 ± 10.68	53.73 ± 10.33	0.26
LVEF < 50% (%)	51 (38.3)	39 (41.1)	12 (31.6)	0.31
**Lipid and metabolic profile before admission**				
Fasting blood glucose (mg/dL)	100.58 ± 22.34	98.38 ± 21.34	106.03 ± 24.05	0.08
Total cholesterol (mg/dL)	211.32 ± 50.9	225.52 ± 45.26	175.82 ± 47.2	<0.001
Triglycerides (mg/dL)	155.4 ± 91.59	162.34 ± 103.11	138.18 ± 49.88	0.17
HDL-C (mg/dL)	48.04 ± 10.83	48.17 ± 11.06	47.71 ± 10.35	0.83
Non-HDL-C (mg/dL)	163.28 ± 48.54	177.35 ± 43.19	128.11 ± 43.46	<0.001
LDL-C (mg/dL)	140.93 ± 41.26	153.85 ± 34.8	108.63 ± 38.67	<0.001
Corrected basal LDL-C (mg/dL) ^#^	161.88 ± 43.43	153.85 ± 34.8	181.96 ± 55.44	0.005
HbA1c (%) *	6.33 ± 1.26	6.09 ± 1.36	6.71 ± 1	0.11
**Lipid and metabolic profile during admission**				
Fasting blood glucose (mg/dL)	117.85 ± 40.67	112.09 ± 32.25	132.24 ± 54.43	0.04
Total cholesterol (mg/dL)	162.11 ± 40.66	164.97 ± 39.95	154.95 ± 42.07	0.2
Triglycerides (mg/dL)	138.02 ± 61.74	136.96 ± 66.98	140.68 ± 46.81	0.75
HDL-C (mg/dL)	39.52 ± 10.02	39.69 ± 10.32	39.08 ± 9.37	0.75
Non-HDL-C (mg/dL)	122.59 ± 37.41	125.27 ± 36.74	115.87 ± 38.73	0.19
LDL-C (mg/dL)	101.71 ± 33.07	105.09 ± 31.31	93.24 ± 36.16	0.06
HbA1c (%)	6.13 ± 1.11	6 ± 1.06	6.44 ± 1.19	0.06
Lipoprotein (a) (mg/dL)	52.74 ± 50.07	48.45 ± 46.44	62.64 ± 57.05	0.16

Abbreviations: GRACE = Global Registry of Acute Coronary Events. HbA1c = glycated hemoglobin. HDL-C = high-density lipoprotein cholesterol. LDL-C = low-density lipoprotein cholesterol. LLT = lipid-lowering therapy. LVEF = left ventricular ejection fraction. ^#^ Corrected basal LDL-C is defined as the LDL-C before admission (in patients without previous LLT) or the corrected LDL-C (in patients with previous LLT). Readers are referred to “Section 2.4” for further details. * HbA1c measurement was unavailable in 89 (66.9%) patients before admission.

**Table 2:** Differences in lipid and metabolic profiles before and during admission.

	All Patients (*n* = 133)		No Previous LLT (*n* = 95)		Previous LLT (*n* = 38)		Comparison between LLT Groups
Lipid and Metabolic Profile Variables	Mean Difference ± SD ^†^	*p*-Value ^†^	Mean Difference ± SD ^†^	*p*-Value ^†^	Mean Difference ± SD ^†^	*p*-Value ^†^	*p*-Value ^††^
Fasting blood glucose (mg/dL)	16.96 ± 34.76	<0.001	13.22 ± 28.07	<0.001	26.21 ± 46.63	0.001	0.12
Total cholesterol (mg/dL)	−49.21 ± 43.01	<0.001	−60.55 ± 41	<0.001	−20.87 ± 34.27	0.001	<0.001
Triglycerides (mg/dL)	−17.41 ± 79.25	0.01	−25.38 ± 85.26	0.005	2.5 ± 58.03	0.79	0.07
HDL-C (mg/dL)	−8.52 ± 8.82	<0.001	−8.47 ± 8.95	<0.001	−8.63 ± 8.59	<0.001	0.93
Non-HDL-C (mg/dL)	−40.69 ± 41.4	<0.001	−52.07 ± 39.03	<0.001	−12.24 ± 32.87	0.03	<0.001
LDL-C (mg/dL)	−39.23 ± 34.89	<0.001	−48.76 ± 34.24	<0.001	−15.4 ± 23.4	<0.001	<0.001
LDL-C (mg/dL), compared to corrected basal LDL-C ^#^	−60.18 ± 40.22	<0.001	−48.76 ± 34.24	<0.001	−88.72 ± 40.28	<0.001	<0.001
HbA1c (%) *	0.22 ± 0.44	0.006	0.14 ± 0.28	0.03	0.34 ± 0.61	0.06	0.28

Abbreviations: HbA1c = glycated hemoglobin. HDL-C = high-density lipoprotein cholesterol. LDL-C = low-density lipoprotein cholesterol. LLT = lipid-lowering therapy. ^#^ Corrected basal LDL-C is defined as the LDL-C before admission (in patients without previous LLT) or the corrected LDL-C (in patients with previous LLT). Readers are referred to “Section 2.4” for further details. * HbA1c measurement was unavailable in 89 (66.9%) patients before admission. ^†^ Comparison of lipid and metabolic profile variables before and during admission. ^††^ Comparison of mean differences between no previous LLT and previous LLT groups.

**Table 3:** LLT at discharge.

LLT	Number	Percentage
Statins	131	98.5%
Fluvastatin 80 mg o.d.	1	0.8%
Atorvastatin 20 mg o.d.	1	0.8%
Atorvastatin 40 mg o.d.	15	11.3%
Atorvastatin 60 mg o.d.	3	2.3%
Atorvastatin 80 mg o.d.	49	36.8%
Rosuvastatin 10 mg o.d.	4	3%
Rosuvastatin 15 mg o.d.	1	0.8%
Rosuvastatin 20 mg o.d.	57	42.9%
Ezetimibe 10 mg o.d.	70	52.6%
PCSK9 inhibitors	1	0.8%
Fibrates	3	2.3%

Abbreviations: LLT = lipid-lowering therapy. PCSK9 = proprotein convertase subtilisin/kexin type 9.

## Data Availability

The data presented in this study are available on request from the corresponding authors. The data are not publicly available due to ethical restrictions.

## Supplementary Materials

The following supporting information can be downloaded at: https://www.mdpi.com/article/10.3390/jcm13010127/s1, Table S1: Estimated percentage of LDL-C reduction according to LLT.
