# Supplementary material for: Short-Course High-Intensity Statin Treatment during Admission for Myocardial Infarction and LDL-Cholesterol Reduction—Impact on Tailored Lipid-Lowering Therapy at Discharge
Source: J Clin Med. 2023 Dec 25;13(1):127. doi: 10.3390/jcm13010127 (PMC10780070; doi:10.3390/jcm13010127)
Supplement: Supplementary file 1 [file jcm-13-00127-s001.zip › jcm-2745958-supplementary.pdf]

Journal of Clinical Medicine

MDPI

Supplementary Materials

Short-Course High-Intensity Statin Treatment during Admission for Myocardial Infarction and LDL-Cholesterol Reduction—Impact on Tailored Lipid-Lowering Therapy at Discharge

Víctor Marcos-Garcés [1,2,*], Héctor Merenciano-González [1,2], María Luz Martínez Mas [1], Patricia Palau [1,2,3], Josefa Inés Climent Alberola [4], Nerea Perez [2], Laura López-Bueno [4], María Concepción Esteban Argente [4], María Valls Reig [1], Raquel Muñoz Alcover [1], Inmaculada Pradillas Contreras [1], Ana Arizón Benito [5], Alfonso Payá Rubio [4], César Ríos-Navarro [2], Elena de Dios [6], Jose Gavara [7], Francisco Javier Chorro [1,2,3,6], Juan Sanchis [1,2,3,6] and Vicente Bodi [1,2,3,6,*]

1. Department of Cardiology, Hospital Clinico Universitario de Valencia, 46010 Valencia, Spain; hectormeren@gmail.com (H.M.-G.); mluzmmas@comv.es (M.L.M.M.); patricia.palau@uv.es (P.P.); mvallsr@gmail.com (M.V.R.); raquelalcover@hotmail.com (R.M.A.); inmapracon@yahoo.es (I.P.C.); francisco.j.chorro@uv.es (F.J.C.); sanchis_juafor@gva.es (J.S.)
2. INCLIVA Health Research Institute, 46010 Valencia, Spain; neere_8@hotmail.com (N.P.); cesar.rios@uv.es (C.R.-N.)
3. Department of Medicine, Faculty of Medicine and Odontology, University of Valencia, 46010 Valencia, Spain
4. Department of Rehabilitation, Hospital Clinico Universitario de Valencia, 46010 Valencia, Spain; inesclimentO93@gmail.com (J.I.C.A.); laura.lopez@uv.es (L.L.-B.); lulilloluli@yahoo.es (M.C.E.A.); paya_alf@gva.es (A.P.R.)
5. Hospital Clinico Universitario de Valencia, 46010 Valencia, Spain; arizon_anaben@gva.es
6. Network Biomedical Research Center for Cardiovascular Diseases (CIBER-CV), 28029 Madrid, Spain; elenaddll@gmail.com
7. Centre for Biomaterials and Tissue Engineering, Universitat Politècnica de València, 46022 Valencia, Spain; jose_4_6_90@hotmail.com

* Correspondence: marcos_vic@gva.es (V.M.-G.); vicente.bodi@uv.es (V.B.)

1. Supplementary Tables

Table S1. Estimated percentage of LDL-C reduction according to LLT.

LLT	% reduction in mono-therapy	% reduction in combination with ezetimibe 10mg o.d.
**Rosuvastatin 5mg o.d.**	40	54
**Rosuvastatin 10mg o.d.**	46	58
**Rosuvastatin 20mg o.d.**	51	62
**Rosuvastatin 30mg o.d.**	54	65
**Rosuvastatin 40mg o.d.**	56	66
**Atorvastatin 10mg o.d.**	37	51
**Atorvastatin 20mg o.d.**	43	56
**Atorvastatin 30mg o.d.**	46	58
**Atorvastatin 40mg o.d.**	49	61
**Atorvastatin 80mg o.d.**	55	65
**Simvastatin 5mg o.d.**	24	41
**Simvastatin 10mg o.d.**	28	45
**Simvastatin 20mg o.d.**	33	48

**Simvastatin 40mg o.d.**	39	53
**Simvastatin 80mg o.d.**	44	57
**Pravastatin 10mg o.d.**	20	38
**Pravastatin 20mg o.d.**	26	43
**Pravastatin 40mg o.d.**	29	45
**Pravastatin 80mg o.d.**	35	50
**Pitavastatin 1mg o.d.**	32	48
**Pitavastatin 2mg o.d.**	36	51
**Pitavastatin 4mg o.d.**	43	56
**Fluvastatin 20mg o.d.**	20	38
**Fluvastatin 40mg o.d.**	24	41
**Fluvastatin 80mg o.d.**	34	49
**Lovastatin 10mg o.d.**	20	38
**Lovastatin 20mg o.d.**	28	45
**Lovastatin 40mg o.d.**	31	47
**Lovastatin 80mg o.d.**	40	54
**Ezetimibe 10mg o.d.**	23	-
**Evolocumab 140mg o.d.**	+61% reduction	+70% reduction
**Alirocumab 75mg o.d.**	+53% reduction	+64% reduction
**Alirocumab 150mg o.d.**	+62% reduction	+71% reduction

Abbreviations: LDL-C = low-density lipoprotein cholesterol. LLT = lipid-lowering therapy.
